# Supplementary material for: PfAgo-Based Zika Virus Detection
Source: Viruses. 2024 Mar 30;16(4):539. doi: 10.3390/v16040539 (PMC11054744; doi:10.3390/v16040539)
Supplement: Supplementary file 1 [file viruses-16-00539-s001.zip › viruses-2914105-supplementary.pdf]

Supplementary data

Supplementary Materials and Methods

1. Oligos, enzymes, and reagents

The primers, gDNAs, ssDNA (MB-ZIKV) was synthesized by Sangon Biotech (Shanghai, China). Moreover, all the nucleic acid sequences, including the ZIKV and DENV1 conserved region, the nonspecific RV(GenBank accession number: AF435866.1) E1 and CMV(GenBank accession number: KF490085.1) gL-UL115 DNA are summarized in Supplementary Table 1. T4 polynucleotide kinase (PNK) was purchased from New England Biolabs (MA, United States). 2×HieffTM PCR Master Mix and Hieff qPCR SYBR Green Master Mix obtained from Yeason Biotech (Shanghai, China) were used to measure the PCR and qPCR reaction assays. The HiScriptII Q RT SuperMix for qPCR purchased from Vazyme (Shanghai, China) was validated for cDNA synthesis.

2. PCR and qRT-PCR assay

For amplification of ZIKV NS5 PCR product target DNA used in PAND detection, the primers NS-F/R and ZIKV-qPCR-F/R were used for PCR assays and qRT-PCR assay. For qPCR assay, the cycler condition program consisting of initial denaturation at 95 °C for 5 min; then 40 cycles of 95 °C for 10 s, 60 °C for 30 s; followed by extension at 60 °C for 1 min. All measurements were performed in triplicates.

Name	Sequence（5' to 3'）
gn (gMB-ZIKV-NS5)	ATGTGCAAACCTATCA
gn1 (gMB1-ZIKV-NS5)	CTGAGGGCATGTGCAA
gn2 (gMB2-ZIKV-NS5)	ATTCAAGAACCTGAGG
MB-ZIKV-NS5	CGCACC TGATAGGTTTGCACAT GGTGCG
MB1-ZIKV-NS5	CGCACC TTGCACATGCCCTCAG GGTGCG
MB2-ZIKV-NS5	CGCACC CCTCAGGTTCTTGAAT GGTGCG
gt	AGCCAATTGATGATAG
gf	GTTTGCACATGCCCTC
gr	ACCTGAGGGCATGTGC
ZIKV-NS5-F	GAGATGCAAGACTTGTGGCTG
ZIKV-NS5-R	GTGGTGGGAGCAAAACGGAA
pUC18-ZIKV-F	TTTTGCTCCCACCACGCTCGAATTCGTAATCATGGTCATA
pUC18-ZIKV-R	CAAGTCTTGCATCTCTAGAGTCGACCTGCAGGCATGCA
pEGFP-ZIKV-F	TCGAGCTCAAGCTTCGAGATGCAAGACTTGTGGCTG
pEGFP-ZIKV-R	TCGACTGCAGAATTCGTGGTGGGAGCAAAACGGAA
pEGFP-F	GAATTCTGCAGTCGACGGTAC
pEGFP-R	GAAGCTTGAGCTCGAGATCTG
pEGFP-DENV-F	TCGAGCTCAAGCTTCGAGAGGCTGAAAAGAATGGCAATC
pEGFP-DENV-R	TCGACTGCAGAATTCTGGTGTGAACAGAAAGGCAC
ZIKV-qPCR-F	CGAATGGCAGTCAGTGGAGA

ZIKV-qPCR-R	TGTCCCATCCAGTTGAGGGT
DENV-F	GAGAGGCTGAAAAGAATGGCAATC
DENV-R	TGGTGTGAACAGAAAGGCAC
RV E1-F	GCCGGCGTGTCGTGCAA
RV E1-R	ACGCTCCGGCGTGGC
CMV gL-UL115-F	AACTCCGTGCTGTTGGAC
CMV gL-UL115-R	AACACGTGTTCCGTGAAGATG
ZIKV target	GAGATGCAAGACTTGTGGCTGCTGCGGAGGTCAGAGAAAGTGACC<br>AACTGGTTGCAGAGCAACGGATGGGATAGGCTCAAACGAATGGCA<br>GTCAGTGGAGATGATTGCGTTGTGAAGCCAATTGATGATAGGTTTG<br>CACATGCCCTCAGGTTCTTGAATGATATGGGAAAAGTTAGGAAGG<br>ACACACAAGAGTGGAAACCCTCAACTGGATGGGACAACTGGGAAG<br>AAGTTCCGTTTTGCTCCCACCAC
DENV target	GAGAGGCTGAAAAGAATGGCAATCAGTGGAGATGACTGTGTGGTG<br>AAACCAATCGATGACAGATTTGCAACAGCCTTAACAGCTTTGAATG<br>ACATGGGAAAGGTAAGAAAAGACATACCGCAATGGGAACCTTCAA<br>AAGGATGGAATGATTGGCAACAAGTGCCTTTCTGTTCACACCA
RV E1	GCCGGCGTGTCGTGCAACGTCACCACTGAACACCCGTTCTGCAACA<br>CGCCGCACGGACAACTCGAGGTCCAGGTCCCGCCCGACCCTGGGG<br>ACCTGGTTGAGTACATTATGAATTACACCGGCAATCAGCAGTCCCG<br>GTGGGGCCTCGGGAGCCCGAATTGTCATGGCCCCGATTGGGCCTCC<br>CCGGTTTGCCAACGCCATTCCCCTGACTGCTCGCGGCTTGTGGGGG<br>CCACGCCGGAGCGT
CMV gL-UL115	AACTCCGTGCTGTTGGACGATGCTTTCCTGGACACTCTGGCCCTGC<br>TGTACAACAATCCGGATCAATTACGGGCCCTGCTGACGCTGTTGAG<br>CTCGGACACAGCGCCGCGCTGGATGACGGTGATGCGCGGCTACAG<br>CGAGTGCGGCGATGGCTCGCCGGCCGTGTACACGTGCGTGGACGA<br>CCTGTGCCGCGGCTACGACCTTACGCGACTGTCATACGGGCGCAGC<br>ATCTTCACGGAACACGTGTT

Table S1. Oligonucleotides and primers used in this report

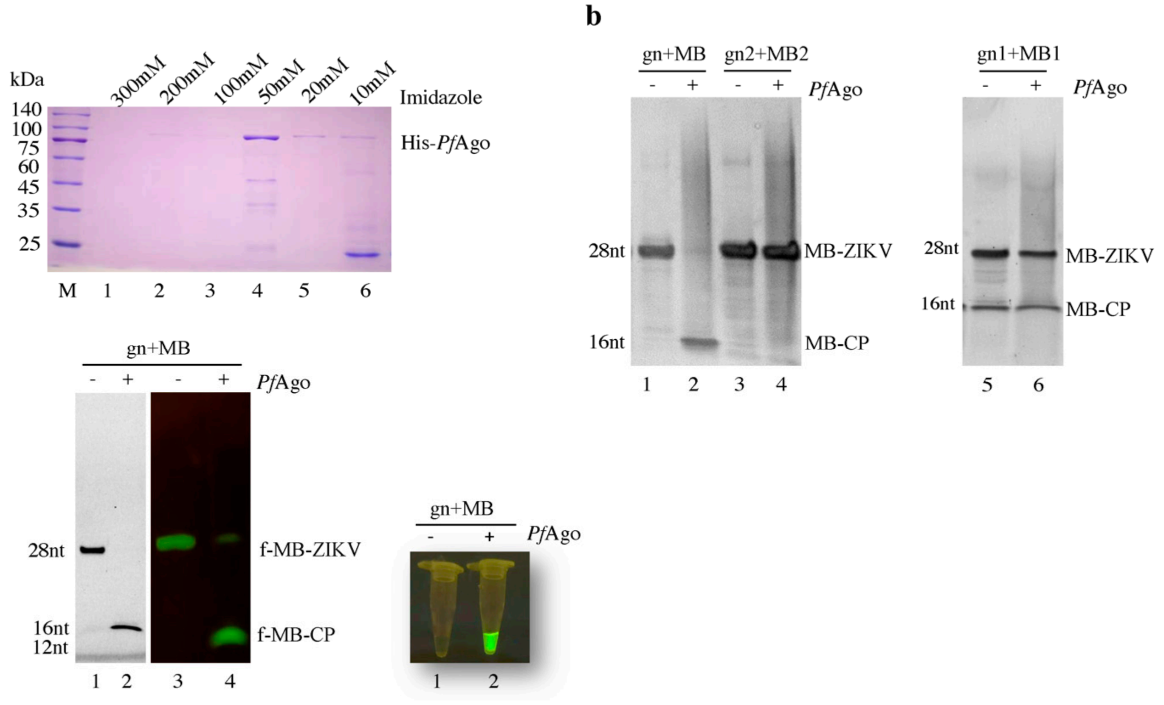

Figure S1. Establishment of the *Pf*Ago-mediated Zika viral nucleic acid detection system

(a). SDS-PAGE assay of the purified His-*Pf*Ago fusion protein. M: Protein ladder. **(b).** Identification of ZIKV target MB for gn mediated *Pf*Ago-specific cleavage was indicated on 20% TBE-PAGE electrophoresis. **(c).** gn mediated *Pf*Ago-specific cleavage of f-MB-ZIKV was indicated on 20% TBE-PAGE electrophoresis with white light (Left panel, lane1-2), Blue light (Left panel, 3-4) or imaged in the tube using Blue-Light Transilluminators (Right panel, 1-2). The size of the targets (MB-ZIKV/f-MB-ZIKV, 28 nt), gDNA (gn/gn1/gn2,16 nt), and band of cleavage products (MB-ZIKV CP,16 nt /f-MB-ZIKV CP,12 nt) are shown on the left. CP: Cleavage products, gDNA: guide DNA, nt: nucleotide.

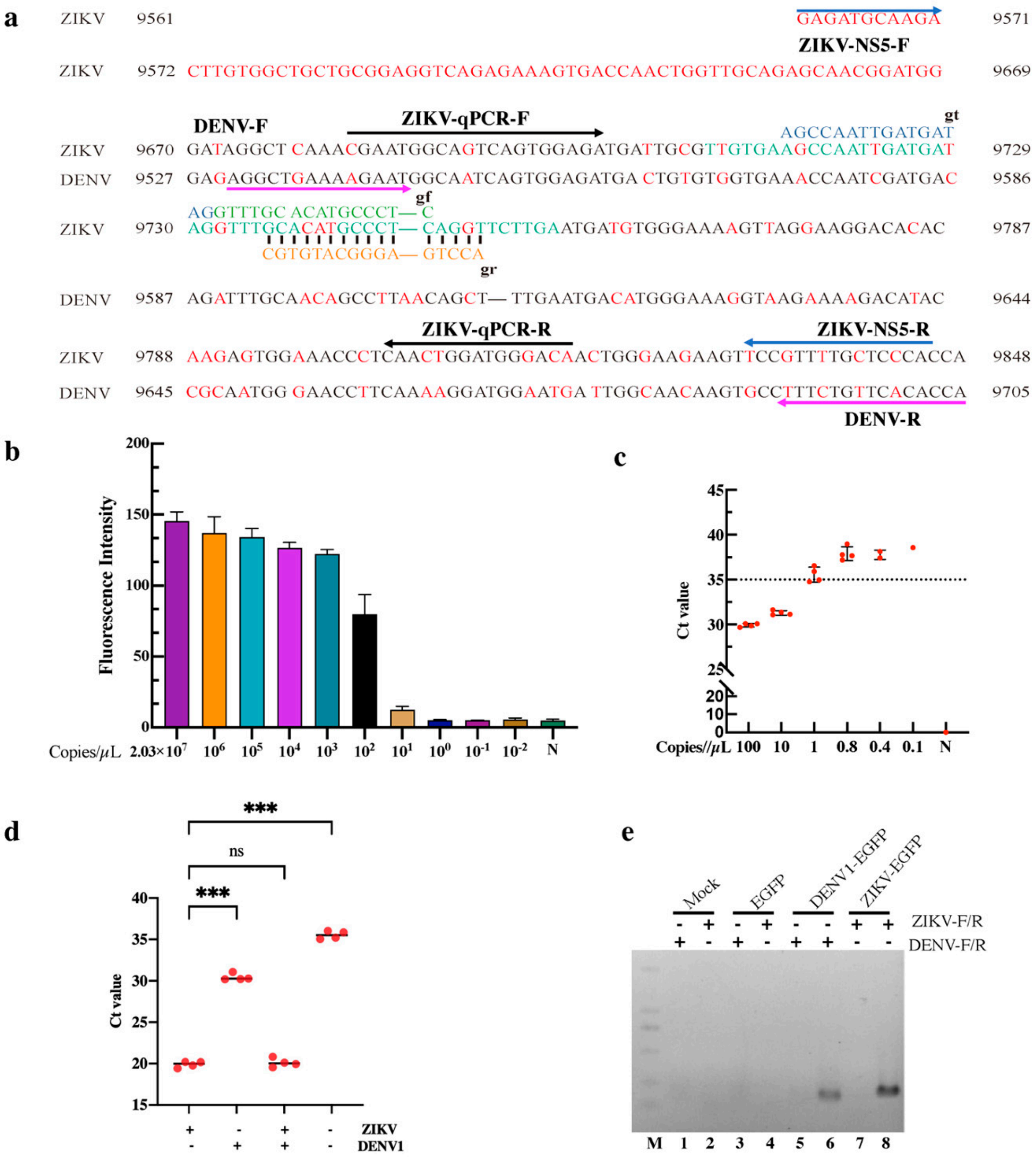

Figure S2. The ZIKV Detection with qRT-PCR or RT-PCR. (a). Sequence alignment of ZIKV and DENV1 target region for ZIKV-PAND and qRT-PCR. The unpaired nucleotide bases between gDNA (gt/gf/gr) and DENV1 were indicated in red. The location of ZIKV-qPCR primers, ZIKV and DENV1 RT-PCR primers indicated in black, red, and blue arrows respectively were also shown. **(b).** The MDC analysis of the three gDNA mediated ZIKV-PAND with PCR. A final concentration of template pUC-ZIKV from 6.73×10^{8} aM to 6.73×10^{-1} aM (2.03×10^{-2} copies) was added. **(c).** MDC of ZIKV qRT-PCR assay. **(d).** The specificity of ZIKV Detection with qPCR. **(e).** Analysis of sample simulation with ZIKV RT-PCR. The primers and samples from transfection with EGFP, DENV1-EGFP, ZIKV-EGFP were shown. Error bars represent mean ± SD, where n = 4 replicates. Student's t test, ***P < 0.001. n.s, not significant.

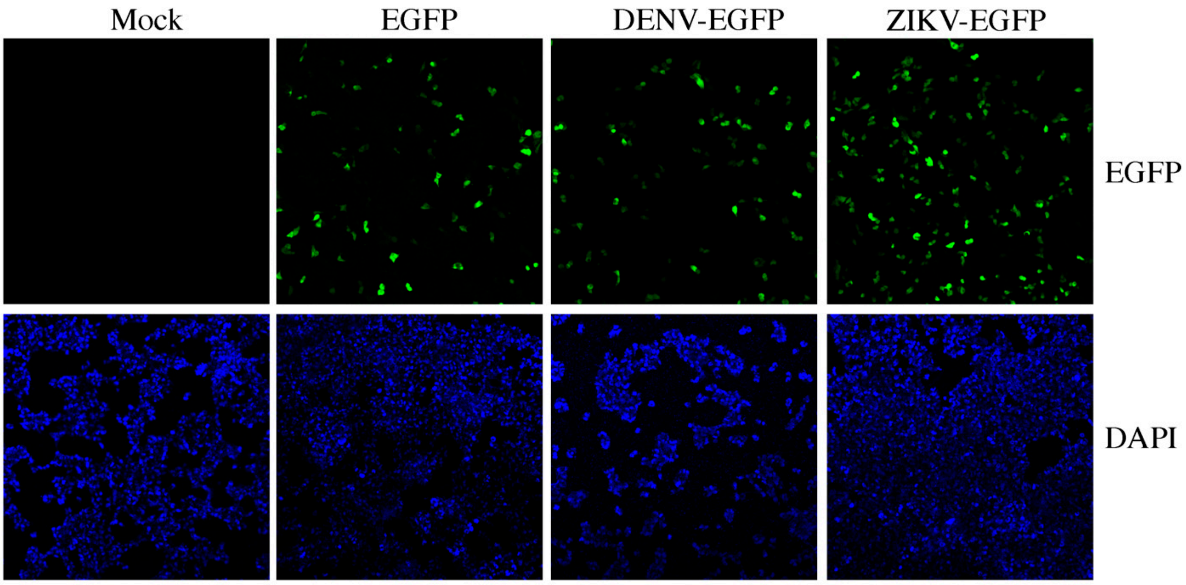

Figure S3. Analysis of EGFP expression with CMV-promoter driven ZIKV-EGFP/DENV1-EGFP transfection. HEK293T cells were transfected with CMV-promoter driven constructs (ZIKV-EGFP/DENV1-EGFP) transcribing partial NS5 targeted RNA of ZIKV and DENV1 individually, as indicated on the top, and stained 48h later with DAPI. Representative images are shown. All images were taken with a 10× objective.

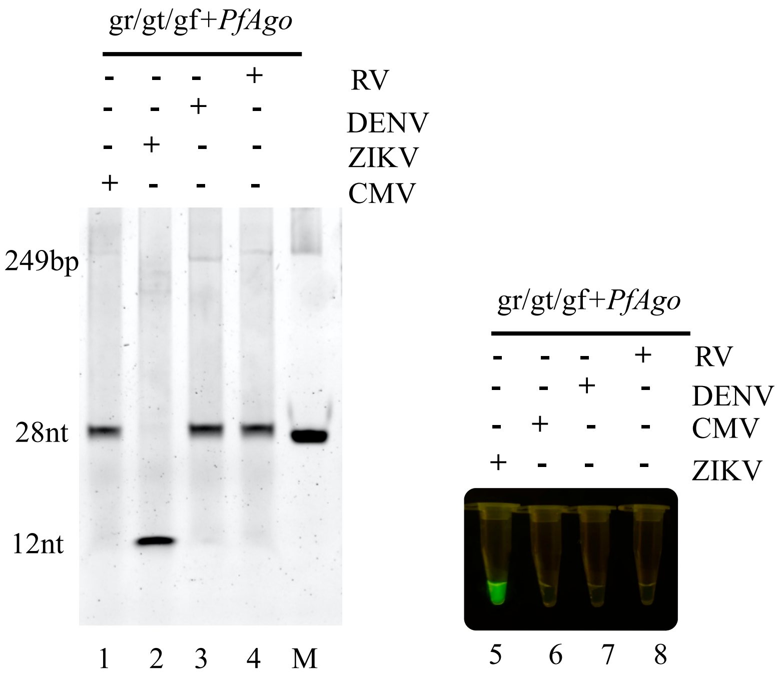

Figure S4. gr/gt/gf mediated *Pf*Ago-specific cleavage of f-MB-ZIKV with nonspecific viral DNA was performed as described in the specificity of ZIKV-PAND Detection from Materials and Methods. The results were indicated on 20% TBE-PAGE electrophoresis with white light (Left panel, lane1-4) or imaged in the tube using Blue-Light Transilluminators (Right panel, 5-8). M: Marker (f-MB-ZIKV), RV, DENV and CMV DNA was indicated as nonspecific viral DNA. The size of the targets (f-MB-ZIKV, 28 nt), gDNA (gr/gt/gf,16 nt) are shown on the left.

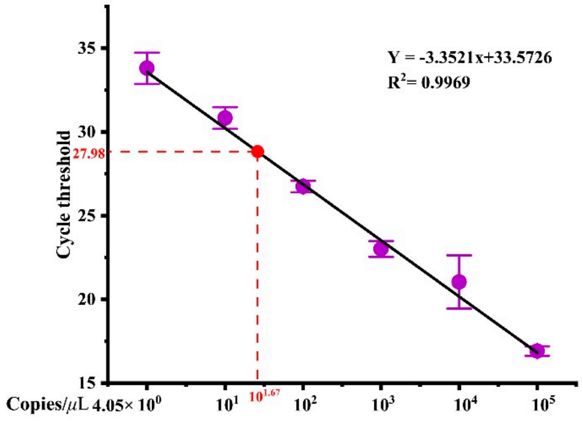

Figure S5. Standard curve for the quantification of viral copy number by real-time PCR. The used ten-fold dilutions ranged from 4.05 × 10^5 to 4.05 × 10^0 copies per μL and were plotted as X-axis. CT values are indicated on Y-axis. $10^{1.67}$ copies/μL of viral RNA genome was calculated. The standard curve parameters calculation was given above the curve.

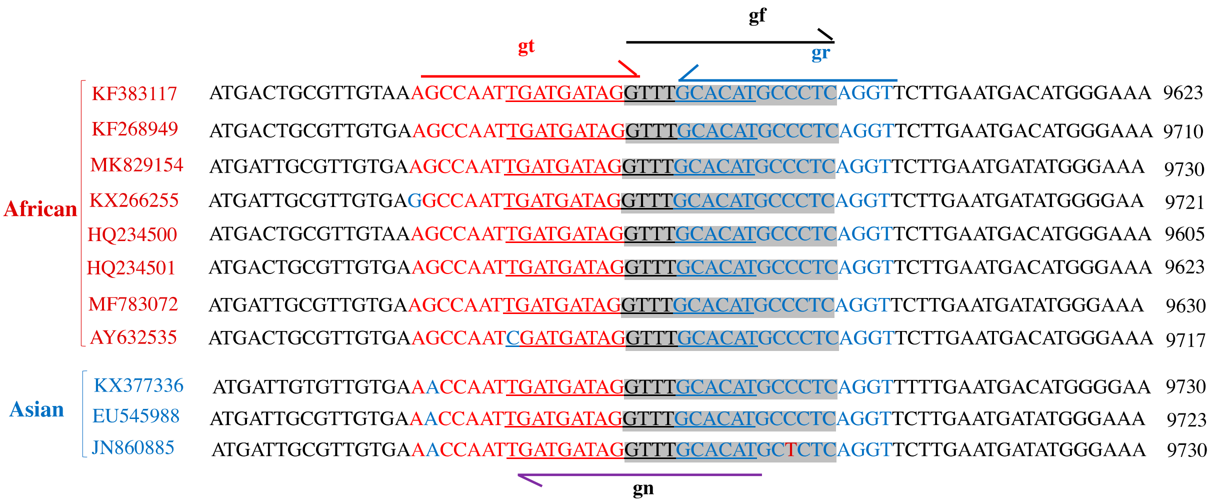

Figure S6. Analysis of partial Zika virus NS5 nucleotide sequence alignments.

The African lineages are indicated by blue color. The Asian lineages are indicated by red color. The guide DNAs such as gt, gf, gr and gn are shown in red, gray, blue color, and underlined, respectively. The numbers indicate the end position of these nucleotide sequences in the ZIKV genome. GenBank accession number of ZIKV was indicated on the left.
